# Ribosome Biogenesis and Translational Control in Skeletal Muscle Atrophy and Hypertrophy: Mechanisms and Therapeutic Perspectives

**DOI:** 10.3390/biom16030406

**Published:** 2026-03-10

**Authors:** Miaomiao Xu, Xiaoguang Liu

**Affiliations:** 1 College of Physical Education, Guangdong University of Education, Guangzhou 510800, China; xumiaomiao@gdei.edu.cn; 2 College of Sports and Health, Guangzhou Sport University, Guangzhou 510500, China; 3 Research Center for Innovative Development of Sports and Healthcare Integration, Guangzhou Sport University, Guangzhou 510500, China

**Keywords:** ribosome biogenesis, translational control, skeletal muscle atrophy, ribosome heterogeneity, therapeutic strategies

## Abstract

Maintenance of skeletal muscle mass is essential for mobility, metabolic homeostasis, and clinical outcomes across a wide spectrum of physiological and pathological conditions. While muscle atrophy and hypertrophy have traditionally been interpreted through upstream anabolic–catabolic signaling and proteolytic pathways, accumulating evidence indicates that ribosome biogenesis and translational control represent rate-limiting determinants of muscle plasticity. However, this regulatory layer remains insufficiently integrated into current models of muscle adaptation and disease. In this review, we synthesize recent advances in ribosomal RNA transcription, ribosomal protein dynamics, and translational regulation in skeletal muscle, with particular emphasis on signaling networks governed by mTORC1, c-Myc, AMPK, and FOXO. We highlight ribosome biogenesis as a central hub linking mechanical loading, nutrient availability, inflammatory stress, and metabolic status to protein synthesis capacity. Evidence from human and animal studies demonstrates that impaired ribosome production and translational efficiency precede and predict muscle atrophy in disuse, aging, cancer cachexia, and chronic disease, whereas ribosome expansion is a prerequisite for sustained hypertrophy. Beyond quantitative regulation, we discuss the emerging concept of ribosome heterogeneity as a qualitative layer of translational control that may enable selective mRNA translation during muscle growth, stress adaptation, and degeneration. We further examine ribosome–mitochondria crosstalk as a critical but underexplored mechanism coordinating anabolic capacity with cellular energetics. Finally, we outline therapeutic implications, highlighting exercise, nutritional strategies, and indirect pharmacological interventions that preserve ribosomal competence, and propose ribosome-based biomarkers as promising tools for precision management of muscle-wasting disorders. Collectively, this review positions ribosome biology as a translationally relevant framework bridging molecular mechanisms with therapeutic perspectives in skeletal muscle atrophy and hypertrophy.

## 1. Introduction

Skeletal muscle accounts for approximately 40% of total body mass and plays essential roles in locomotion, posture maintenance, and metabolic homeostasis, particularly in glucose and lipid metabolism [1,2,3]. Owing to its remarkable plasticity, skeletal muscle continuously adapts to diverse physiological and pathological stimuli, including mechanical loading, physical inactivity, aging, chronic disease, and cancer [3,4,5,6]. Disruption of this adaptive capacity leads to muscle atrophy, a condition characterized by reduced muscle mass and strength that contributes to frailty, metabolic dysfunction, impaired quality of life, and increased mortality [7,8,9].

Muscle atrophy is prevalent in aging populations, prolonged immobilization, chronic diseases (e.g., chronic kidney disease), and cancer cachexia [4,5,6,10,11]. In contrast, muscle hypertrophy represents an adaptive anabolic response to resistance exercise (RE), mechanical overload, and adequate nutritional support [12,13,14]. At the cellular level, these opposing phenotypes are determined by the balance between protein synthesis and protein degradation [15,16,17]. While extensive research has focused on upstream anabolic–catabolic signaling pathways and proteolytic systems, these mechanisms alone do not fully explain the heterogeneity of muscle adaptive responses observed across individuals, conditions, and interventions [15,17].

Ribosomes are the cellular machinery responsible for translating messenger RNA into protein and therefore directly determine the protein synthesis capacity of skeletal muscle [18]. Ribosome biogenesis is a highly energy-demanding and tightly regulated process involving ribosomal RNA (rRNA) transcription, ribosomal protein (RP) synthesis, and assembly of functional ribosomal subunits [19,20,21]. Emerging evidence suggests that ribosome biogenesis is not merely a downstream consequence of anabolic signaling, but rather a rate-limiting determinant of skeletal muscle plasticity across both hypertrophic and atrophic conditions [22,23]. Impaired ribosome production and reduced translational capacity have been observed in disuse and disease settings and may precede overt muscle loss, whereas sustained ribosome expansion is required for long-term hypertrophy [23,24,25].

Ribosome biogenesis is controlled by an integrated signaling network that senses mechanical, nutritional, energetic, and inflammatory cues. Anabolic pathways, particularly mechanistic target of rapamycin complex 1 (mTORC1) and c-Myc, promote rRNA transcription and ribosomal protein synthesis, thereby enhancing translational capacity [26,27,28,29]. In contrast, catabolic signals such as AMP-activated protein kinase (AMPK) activation, Forkhead box O (FOXO) transcription factors, and chronic inflammation can suppress ribosome production and nucleolar function [15,30,31]. Age-related anabolic resistance further limits ribosomal responsiveness to exercise and nutrition, contributing to the development of sarcopenia [22,32,33].

Beyond quantitative changes in ribosome abundance, recent studies have introduced the concept of ribosome heterogeneity, suggesting that variations in ribosomal protein composition and rRNA modifications may confer selective translational control [34,35,36]. This emerging paradigm has begun to gain experimental support in striated tissues and skeletal muscle, including evidence that rRNA modification landscapes and ribosome-associated features can shift with hypertrophic stimuli [37,38,39]. However, ribosome biology remains underrepresented in many muscle-focused overviews, which have largely emphasized upstream signaling pathways or proteolytic mechanisms without fully integrating ribosome-centered regulation [15,17].

In this review, we provide a comprehensive synthesis of current knowledge on ribosome biogenesis and translational control in skeletal muscle. We integrate molecular mechanisms regulating rRNA transcription, ribosomal protein dynamics, and translational efficiency with evidence from human and animal studies of muscle atrophy and hypertrophy [19,22,23]. We further discuss emerging concepts such as ribosome heterogeneity and ribosome–mitochondria crosstalk, and highlight translational implications for exercise, nutritional, and pharmacological strategies relevant to muscle-wasting disorders [12,13,40,41,42,43,44].

## 2. Methods

To provide an integrative overview of ribosome biogenesis and translational control in skeletal muscle, we conducted a comprehensive literature survey across PubMed, Web of Science, Google Scholar, and EMBASE. The search covered studies published between January 2020 and December 2025 and focused on key terms related to ribosome biology and skeletal muscle adaptation, including ribosome biogenesis, ribosomal RNA, ribosomal proteins, muscle atrophy and hypertrophy, protein synthesis and turnover, skeletal muscle regeneration, mTOR and AMPK signaling, autophagy, sarcopenia, aging-related muscle dysfunction, exercise-induced muscle adaptation, and nutritional interventions. Boolean operators were applied to optimize retrieval of relevant literature. In addition, reference lists of relevant review articles were screened to identify additional studies of interest.

The literature considered in this review primarily comprised peer-reviewed articles published in English, including human and animal studies such as randomized controlled trials, observational and cohort studies, and preclinical experimental investigations. Emphasis was placed on studies addressing muscle atrophy associated with aging, disuse, chronic disease, and cancer cachexia, as well as hypertrophic or protective adaptations induced by exercise and nutritional interventions. Studies focusing exclusively on isolated in vitro systems without in vivo relevance, non-peer-reviewed materials, and publications not directly related to the molecular regulation of skeletal muscle mass were not prioritized.

Although the review was restricted to English-language publications, we acknowledge that relevant studies published in other languages may exist. However, as many non-English articles provide English abstracts, the overall impact of this limitation on the scope of the review is expected to be limited.

**Figure 1:**
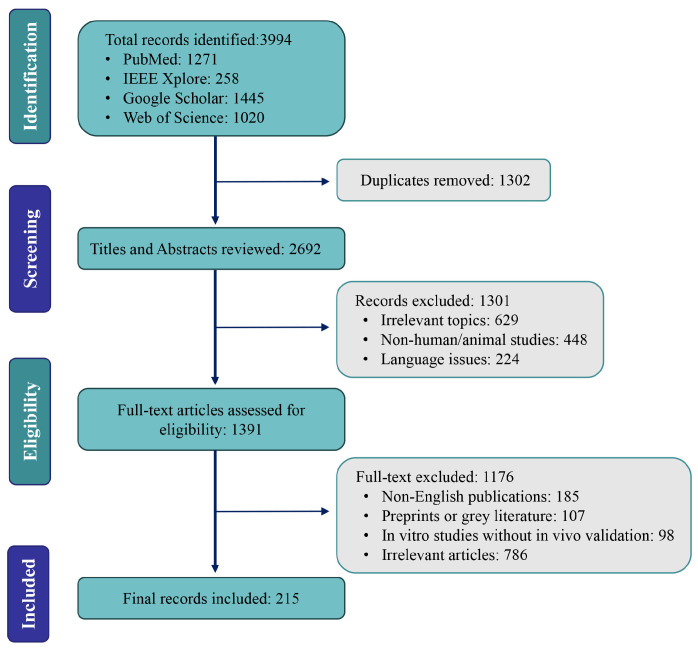
Literature selection flow diagram.

The identified literature was screened based on titles and abstracts, followed by full-text evaluation where appropriate, to ensure relevance to the objectives of this review. This narrative synthesis approach was designed to integrate mechanistic insights from molecular, physiological, and translational studies, rather than to perform a formal systematic review or meta-analysis. For illustrative purposes, an overview of the literature selection process is provided in Figure 1.

## 3. Ribosome Biogenesis in Skeletal Muscle: Molecular Basis

Ribosome biogenesis, recognized as one of the most energetically demanding processes within eukaryotic cells [45], directly dictates the translational capacity of skeletal muscle [22]. Given that muscle fibers are constantly adapting to diverse stimuli such as exercise, disuse, and various disease states, the regulation of ribosome production must be exquisitely dynamic [5]. This intricate process is underpinned by four interconnected regulatory layers: rRNA transcription [46], the synthesis of RPs coupled with their nucleolar assembly [47], a complex network of signaling pathways [19], and the crucial balance between overall translational capacity and efficiency [48].

### 3.1. rRNA Transcription and Processing

The genesis of ribosomes fundamentally commences with rRNA transcription. RNA polymerase I (Pol I) is responsible for synthesizing the 47S pre-rRNA, which subsequently undergoes processing to yield the 18S, 5.8S, and 28S rRNAs [49]. Concurrently, RNA polymerase III (Pol III) transcribes the 5S rRNA, a crucial component for the assembly of the large ribosomal subunit [50]. Post-transcriptional modifications, including methylation and pseudouridylation, are then introduced to these rRNAs [51,52]. These modifications are precisely guided by small nucleolar RNAs (snoRNAs) and are critical for ensuring the structural integrity, stability, and functional fidelity of the mature ribosome [53].

Within skeletal muscle, rRNA transcription demonstrates a high degree of responsiveness to various physiological and pathological stimuli [54]. The magnitude and kinetics of this response are strictly modulated by the mode and duration of mechanical loading. In laboratory animals, chronic mechanical overload (MOV), commonly induced by synergist ablation, triggers a marked expansion of the ribosomal pool. Experimental studies in rodent models demonstrate that mechanical overload rapidly activates RNA Pol I-mediated rDNA transcription and ribosome biogenesis, leading to a substantial increase in total RNA content within days of loading [23,55,56]. This rapid accumulation of ribosomes often precedes detectable increases in muscle fiber cross-sectional area, indicating that expansion of translational capacity—the total number of ribosomes available for protein synthesis—is a prerequisite for maximal compensatory hypertrophy [23,25].

In humans, the response to mechanical loading is more nuanced. While acute RE prompts a transient surge in 47S pre-rRNA levels within hours [57], chronic RE training is required to achieve a stable elevation in the basal ribosomal pool [57,58]. Notably, the capacity to upregulate ribosome biogenesis appears to be a key determinant of hypertrophic responsiveness. Individuals classified as “high responders” to resistance training exhibit greater expansion of rRNA and ribosome-related transcripts compared with “low responders,” highlighting ribosome biogenesis as a critical contributor to inter-individual differences in anabolic adaptation [24,25].

**Figure 2:**
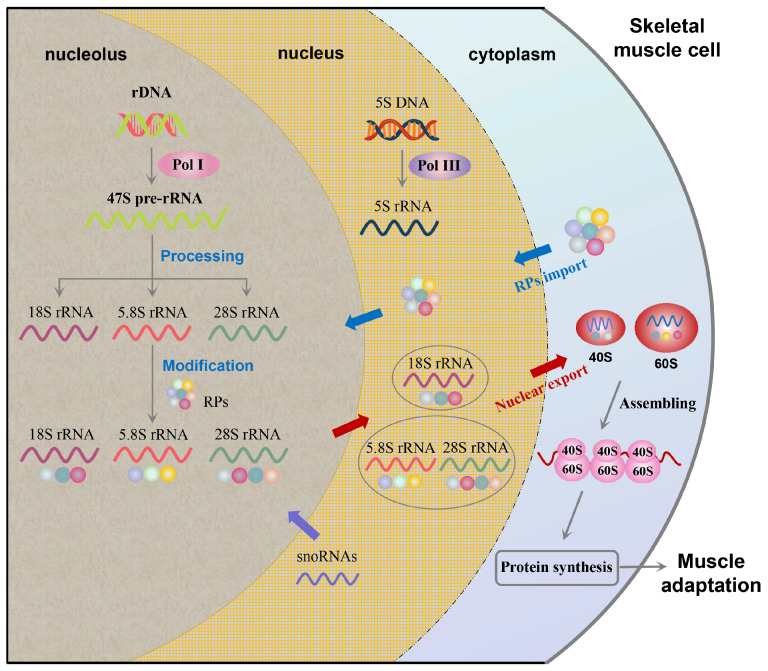
Overview of ribosome biogenesis in skeletal muscle. This schematic illustrates the sequential process of ribosome biogenesis in skeletal muscle cells. In the nucleolus, rDNA is transcribed by Pol I to produce 47S pre-rRNA, which is processed into 18S, 5.8S, and 28S rRNAs and modified by RPs under the guidance of snoRNAs. Meanwhile, Pol III transcribes 5S DNA to generate 5S rRNA. The 18S rRNA associates with RPs to form the 40S subunit, whereas 5S, 5.8S, and 28S rRNAs combine with RPs to form the 60S subunit. Both subunits are exported to the cytoplasm, where they assemble into functional ribosomes that drive protein synthesis and support muscle adaptation. Note: Pol I, RNA polymerase I; Pol III, RNA polymerase III; rDNA, ribosomal DNA; rRNA, ribosomal RNA; RP, ribosomal protein; snoRNA, small nucleolar RNA. Blue arrows indicate RP import; red arrows indicate nuclear export; purple arrows indicate snoRNA-guided modifications essential for ribosome stability and function; gray arrows indicate the direction of molecular assembly and protein synthesis.

Furthermore, ribosome biogenesis plays a pivotal role during muscle regrowth following periods of atrophy. Upon reloading after disuse or immobilization, the rapid reactivation of Pol I-mediated transcription serves as a critical compensatory mechanism to restore proteostasis and muscle mass [23]. Conversely, conditions of disuse or cachexia lead to a sharp suppression of transcription [5]. This transcriptional downregulation often precedes macroscopically measurable muscle loss [59], with immobilized patients showing diminished pre-rRNA expression that correlates closely with compromised translational capacity [60]. It is crucial to note, however, that not every acute surge in rRNA transcription necessarily translates into long-term muscle hypertrophy [24]. This observation suggests that rRNA transcription must be tightly integrated with downstream translational efficiency and the coordinated assembly of ribosomal proteins to fully elucidate the mechanisms of muscle adaptation [22]. The overall process of ribosome biogenesis, from rRNA transcription to subunit assembly and export, is summarized in Figure 2.

### 3.2. Ribosomal Protein Synthesis and Nucleolar Assembly

Approximately 80 distinct RPs are cytoplasmically synthesized, subsequently imported into the nucleolus, and precisely assembled with rRNAs to form the 40S and 60S ribosomal subunits [20]. As depicted in Figure 2, this intricate assembly process, involving the precise integration of RPs with various rRNA components, is crucial for the formation of functional ribosomes. This intricate assembly process necessitates stringent stoichiometric control and is vigilantly monitored by chaperone-mediated quality control mechanisms to ensure fidelity [21].

Resistance training has been shown to upregulate the expression of numerous RP genes [61], while conversely, conditions such as immobilization or systemic illness lead to their suppression [5]. Proteomic investigations have further revealed the preferential induction of specific RPs, such as ribosomal protein L3 (RPL3) and ribosomal protein S6 (RPS6), during muscle hypertrophy [25,38,62]. This observation introduces the intriguing possibility of specialized ribosomes, potentially tailored to facilitate muscle growth [39]. Nevertheless, whether these findings genuinely represent “ribosome specialization” or merely reflect a broader global transcriptional activation within the muscle remains a subject of ongoing debate [34].

From a translational viewpoint, disruptions in RP assembly can result in the formation of ribosomes that, despite appearing structurally intact, exhibit functional impairment [63]. Such compromised ribosomes may consequently contribute to the anabolic resistance observed in contexts of aging or various disease states [22].

### 3.3. Signaling Regulation of Ribosome Biogenesis

The intricate process of ribosome biogenesis is governed by a complex interplay between anabolic and catabolic signaling pathways [27,31]. On the anabolic side, the mTORC1 promotes rRNA synthesis by activating RNA Pol I through transcription initiation factor 1A (TIF-1A) and upstream binding factor (UBF) [64]. Concurrently, mTORC1 enhances RP translation via the regulation of ribosomal protein S6 kinase 1 (S6K1) and eukaryotic translation initiation factor 4E-binding protein (4EBP) [65,66]. Complementarily, c-Myc acts as a transcriptional amplifier, simultaneously upregulating rRNA and RP genes to boost ribosome output [67].

**Figure 3:**
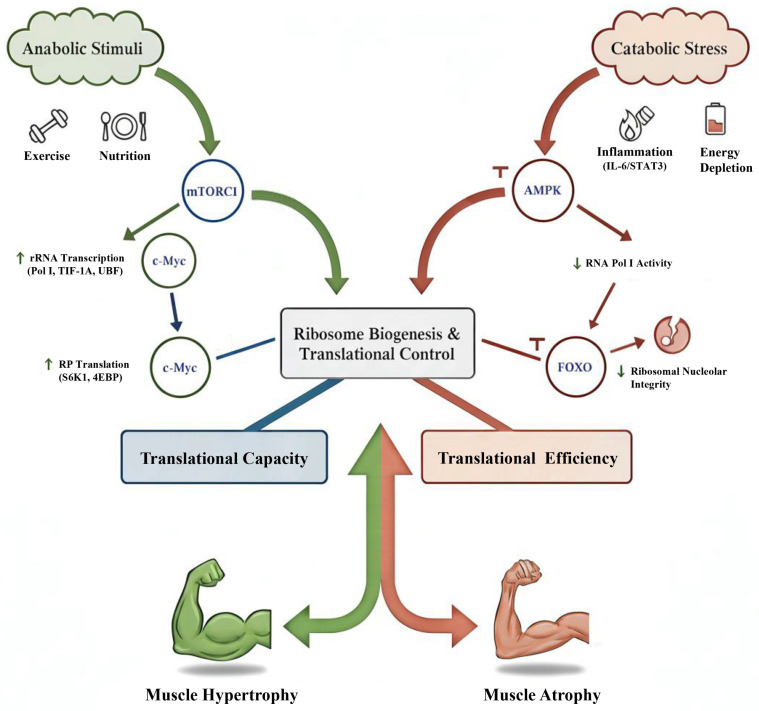
Signaling network regulating ribosome biogenesis and translational control in skeletal muscle. This diagram illustrates the intricate interplay of anabolic and catabolic signaling pathways governing ribosome biogenesis and translational control in skeletal muscle. Anabolic stimuli (e.g., exercise, nutrition) activate mTORC1 and c-Myc (green arrows), promoting rRNA transcription (via Pol I, TIF-1A, UBF) and RP translation (via S6K1, 4EBP) to enhance ribosome biogenesis and translational capacity, leading to muscle hypertrophy. Conversely, catabolic stress (e.g., inflammation, energy depletion) activates AMPK, FOXO, and inflammatory signals (e.g., IL-6/STAT3) (red arrows), which suppress RNA Pol I activity, downregulate ribosomal genes, and compromise nucleolar integrity, thereby diminishing translational efficiency and contributing to muscle atrophy. The balance between these opposing forces precisely orchestrates ribosome function, dictating the anabolic or catabolic fate of muscle. Note: AMPK, AMP-activated protein kinase; c-Myc, cellular myelocytomatosis oncogene; 4EBP, eukaryotic translation initiation factor 4E-binding protein; FOXO, Forkhead box protein O; IL-6, interleukin-6; mTORC1, mechanistic target of rapamycin complex 1; Pol I, RNA polymerase I; rRNA, ribosomal RNA; RP, ribosomal protein; S6K1, ribosomal protein S6 kinase 1; STAT3, signal transducer and activator of transcription 3; TIF-1A, transcription initiation factor 1A; UBF, upstream binding factor. Green arrows and lines indicate activating or promoting effects. Red arrows denote the progression of signaling within the catabolic pathway. T-bar symbols (⊥) indicate inhibitory effects (e.g., AMPK-mediated inhibition of the mTORC1 pathway and FOXO-mediated suppression of ribosome biogenesis).

Conversely, catabolic signals serve to actively curb ribosome production [68]. Under conditions of energetic stress, AMPK effectively suppresses RNA Pol I activity. Furthermore, FOXO transcription factors directly downregulate the expression of ribosomal genes [30]. These actions collectively antagonize the stimulatory effects of mTORC1 and c-Myc, thereby integrating energy status with ribosome synthesis [69]. The comprehensive interplay of these anabolic and catabolic pathways, along with additional regulatory mechanisms, is graphically summarized in Figure 3.

Beyond this conventional anabolic–catabolic dichotomy, recent discoveries reveal additional layers of regulation [70]. For example, inflammatory signaling, notably the interleukin-6/signal transducer and activator of transcription 3 (IL-6/STAT3) pathway active in cancer cachexia, can directly compromise nucleolar integrity, a process independent of mTORC1 activity [71]. Furthermore, AMPK has been demonstrated to not only diminish ribosome abundance but also to induce alterations in ribosomal protein phosphorylation, thereby potentially modifying translational specificity [72]. Cumulatively, these insights suggest that the ribosome dysfunction characteristic of muscle atrophy arises from a dual mechanism: both a reduction in overall biogenesis and an active dismantling process orchestrated by various stress-responsive pathways [73].

### 3.4. Translational Capacity Versus Efficiency

The functionality of ribosomes can be broadly categorized into two aspects: capacity, which refers to the overall abundance of ribosomes, and efficiency, denoting the protein synthesis output per ribosome [22]. Resistance training primarily acts by increasing the ribosomal capacity within muscle cells [25]. In contrast, acute supplementation with amino acids typically boosts translational efficiency through the enhanced activation of initiation and elongation factors [40].

In the context of sarcopenia, both aspects of ribosome function are compromised: there is a noticeable reduction in overall ribosome abundance [74], coupled with a blunted activation of key initiation factors [32]. Conversely, in cachexia, while ribosome numbers might remain relatively stable, translational efficiency often diminishes, largely due to persistent inflammatory stress [75]. Furthermore, ribosome profiling studies conducted in older adults corroborate that impaired translational efficiency, rather than a mere scarcity of ribosomes, frequently constitutes the primary bottleneck in protein synthesis [76].

This critical distinction between ribosome capacity and efficiency helps account for the heterogeneous responses observed in muscle to exercise interventions [14]. For instance, older adults, despite showing an increase in ribosome content following training, may still not achieve significant hypertrophy because their ribosomal efficiency remains compromised [22]. Consequently, it is imperative to consider nutritional and pharmacological strategies as vital adjuncts to exercise in the comprehensive effort to restore optimal translational competence [41].

### 3.5. Ribosome Biogenesis in Satellite Cells (SCs) and Synergistic Regulation of Hypertrophy

Beyond the mature myofibers, recent evidence emphasizes that ribosome biogenesis is a fundamental prerequisite for the activation and function of SCs [77]. When SCs transition from a quiescent state to an activated, proliferative state—typically triggered by RE or muscle injury—they undergo robust metabolic reprogramming [78]. This process is characterized by a significant surge in RNA Pol I-mediated rDNA transcription and ribosomal assembly [23], which is essential to meet the massive protein synthesis demands of myogenic differentiation and repair [79].

Furthermore, the regulation of skeletal muscle hypertrophy is increasingly viewed as a synergistic coordination between ribosome biogenesis within existing fibers and the recruitment of SCs [58]. While acute increases in muscle mass may rely predominantly on expanding the translational capacity (ribosome abundance) of existing myonuclei [25], sustained and significant hypertrophy necessitates SC-mediated “myonuclear addition.” In this model, SCs provide new transcriptional templates (DNA) that, when integrated into the fiber, further amplify the total ribosomal pool [80]. Thus, the synergy between increased ribosomal machinery and SC-driven myonuclear accretion forms the regulatory basis for maximal anabolic adaptation [22].

## 4. Ribosome Dysfunction in Muscle Atrophy

Skeletal muscle atrophy develops when the rate of protein degradation exceeds that of protein synthesis, ultimately leading to a reduction in muscle mass and compromised contractile function [16]. Although proteolytic systems, including the ubiquitin–proteasome pathway and autophagy, have been thoroughly investigated, mounting evidence now points to the suppression of ribosome biogenesis as an early and critical determinant of atrophy [22]. Diminished rRNA transcription and RP synthesis collectively reduce the muscle’s translational capacity, thereby fostering a catabolic environment that often precedes observable morphological wasting [81]. In the subsequent sections, we will delineate ribosome dysfunction across three principal contexts, namely disuse, disease, and aging, before synthesizing these insights into a cohesive framework.

### 4.1. Disuse-Induced Atrophy

Skeletal muscle disuse atrophy serves as a particularly swift and consistent model for investigating ribosomal suppression [23]. Conditions involving physical inactivity, such as immobilization, extended bed rest, or exposure to microgravity, are known to provoke an acute reduction in ribosome biogenesis [82,83]. Time-course analyses in rodent models of mechanical unloading demonstrate that this suppression is an ultra-early event; 47S pre-rRNA levels exhibit a sharp decline within the first 24 h of unloading, markedly preceding the progressive reduction in total RNA content and ribosomal protein expression observed over 7 to 14 days [84].

From a mechanistic perspective, this mechanical unloading disrupts mTORC1–UBF/TIF-1A signaling pathways [85]. In addition to mTORC1 inhibition, the activation of glycogen synthase kinase-3β (GSK-3β) indicates that activated GSK-3β contributes to the repression of RNA polymerase I-dependent transcription, thereby accelerating the decline in translational capacity [86]. Interestingly, the regulatory role of mTORC1 appears to be stage-dependent during the progression of disuse. Rozhkov et al. [87] reported that during the early phase of unloading (1 day), pharmacological inhibition of mTORC1 with rapamycin unexpectedly prevented the reduction in ribosome biogenesis markers, including 47S pre-rRNA, total RNA, and 18S/28S rRNAs. In contrast, at a later stage of unloading (7 days), rapamycin treatment had no detectable effect on the already suppressed ribosomal output [87]. Together, these findings suggest a complex and time-dependent interplay among mTORC1, GSK-3β, and RNA polymerase I activity in regulating ribosome biogenesis during the onset of muscle atrophy.

Human investigations corroborate these preclinical observations. Astronauts experiencing microgravity for periods ranging from one to six months show substantial decreases (up to 30–40%) in rRNA content, as evidenced by vastus lateralis biopsies [88]. In a similar vein, orthopedic patients subjected to limb immobilization exhibit pronounced downregulation of RP genes within 5 to 10 days [60]. Crucially, both animal and human datasets consistently reveal that ribosomal content and translational capacity are restored during the rehabilitation phase, a process that closely mirrors muscle recovery and regrowth [5]. This restoration depends not only on accelerated biogenesis but also on the suppression of ribophagy (the selective autophagic degradation of ribosomes), which is otherwise activated during unloading to facilitate the rapid clearance of the ribosomal pool [23].

Considering its translational implications, ribosomal activity holds potential as an early and highly sensitive biomarker for assessing muscle plasticity [22]. This could empower clinicians to pinpoint bedridden or immobilized patients who face the highest risk of developing muscle atrophy, and moreover, to effectively gauge the success of various rehabilitative interventions [88,89].

### 4.2. Disease-Associated Atrophy

Muscle atrophy linked to various diseases presents a condition that is heterogeneous in its manifestations yet converges on shared mechanistic underpinnings, prominently featuring ribosomal repression [90]. Although the etiologies are diverse, including cancer, chronic illnesses, and iatrogenic factors, the downregulation of ribosome biogenesis consistently emerges as a common pathway, thereby linking systemic stressors to the progression of muscle wasting [6].

Cancer Cachexia. A key feature of cancer cachexia involves the initiation of STAT3, nuclear factor kappa-light-chain-enhancer of activated B cells (NF-κB), and FOXO signaling by tumor-derived factors and a range of pro-inflammatory cytokines, specifically IL-6, tumor necrosis factor-alpha (TNF-α), and interferon-gamma (IFN-γ) [91,92,93,94]. This cascade ultimately culminates in diminished rRNA transcription and a notable reduction in RP expression [95]. Investigations using experimental models have elucidated a pronounced disruption of nucleolar integrity and a significant impairment of RNA Pol I activity [46,96]. Furthermore, direct examination of muscle biopsies from individuals suffering from cachectic cancer consistently reveals a decreased expression of genes associated with ribosome function, which exhibits a robust inverse correlation with the extent of weight loss and patient survival outcomes [75,97]. Consequently, these data underscore ribosomal dysregulation not merely as a contributor to muscle wasting, but also suggest its promise as a valuable prognostic indicator within the oncological setting [98,99,100].

Chronic Diseases. Chronic kidney disease (CKD) is characterized by the inhibitory effects of uremic toxins and oxidative stress on mTORC1 activity, which subsequently results in diminished rRNA synthesis [101,102]. Similarly, patients with heart failure experience systemic inflammation that blunts anabolic signaling cascades and curtails ribosome production [38,103]. Furthermore, intensive care unit-acquired weakness embodies a complex condition where extended immobilization acts synergistically with inflammation to suppress ribosome biogenesis, thereby significantly expediting muscle atrophy [104,105].

Iatrogenic Factors. Prolonged glucocorticoid administration has been shown to decrease RP gene expression, operating via the glucocorticoid receptor–FOXO signaling pathway, thereby contributing to the development of steroid myopathy [86]. Furthermore, various chemotherapeutic agents, including cisplatin and doxorubicin, compromise nucleolar function through mechanisms involving DNA damage, reactive oxygen species (ROS) accumulation, and mitochondrial stress, which ultimately culminates in impaired ribosome biogenesis [106,107]. Crucially, recent clinical data indicate that ribosomal dysfunction induced by chemotherapy constitutes a fundamental basis for the persistent weakness observed in cancer survivors [18]. This strongly underscores ribosomal stress as a pertinent therapeutic target for supportive care interventions [73].

Taken together, these observations emphasize a crucial mechanistic convergence: various pathological states ultimately lead to ribosomal repression. In terms of clinical translation, modulating ribosome biogenesis emerges as a compelling therapeutic avenue [19]. This approach could encompass anti-inflammatory, nucleolar-protective, and metabolic-supportive strategies, thereby effectively linking advanced molecular insights to the clinical management of muscle wasting disorders [34,46].

### 4.3. Age-Related Atrophy

Sarcopenia, characterized as age-associated muscle atrophy, is fundamentally defined by anabolic resistance—a diminished capacity to elicit hypertrophic responses to both exercise and nutritional cues [77]. When contrasted with younger adults, older individuals consistently demonstrate a muted induction of 47S pre-rRNA and ribosomal protein transcripts subsequent to resistance training, thereby signifying impaired ribosome biogenesis [108]. It is probable that this particular deficiency plays a role in the reduced protein synthesis and restricted muscle accretion commonly noted within aging demographics [109]. The blunted hypertrophic response in aged muscle, often referred to as anabolic resistance, is also closely linked to the functional decline of the SC pool. In younger individuals, RE effectively stimulates SC proliferation and fusion, which sustains long-term muscle growth [110]. In contrast, aging is associated with a decline in satellite cell abundance and impaired activation kinetics in response to mechanical loading, which collectively compromise the regenerative capacity of skeletal muscle and contribute to anabolic resistance in older adults [77,110,111]. This deficiency, combined with a muted induction of 47S pre-rRNA following RE, creates a dual bottleneck—impaired recruitment of new myonuclei and compromised translational capacity—thereby limiting the muscle’s ability to counteract sarcopenia through exercise interventions [23].

Beyond a mere decrease in ribosomal output, growing evidence now highlights qualitative impairments in ribosome biology throughout the aging process [112]. Alterations in ribosomal heterogeneity are observed, encompassing age-dependent shifts in rRNA modifications and ribosomal protein composition, which consequently diminish translational fidelity and overall efficiency [113]. Furthermore, a fragmented nucleolar morphology often develops, indicative of a structural deterioration within the ribosome assembly machinery and compromised nucleolar stress responses [114]. Notably, coexisting conditions like type 2 diabetes, cardiovascular ailments, and chronic inflammatory states demonstrably aggravate ribosomal dysfunction, thereby accelerating the advancement of sarcopenia [115,116].

Considering its translational relevance, diminished ribosomal responsiveness offers a compelling mechanistic basis for the constrained effectiveness of conventional interventions in older adults, including resistance training and protein supplementation [117]. This line of reasoning indicates that ribosome biogenesis might function not merely as a fundamental mechanistic determinant of sarcopenia, but also as an innovative biomarker capable of directing tailored exercise and nutritional approaches within geriatric populations [118].

### 4.4. Integrative Perspective

While their etiologies are distinct, disuse, various diseases, and the aging process exhibit a shared molecular signature, characterized by suppressed ribosome biogenesis, diminished translational capacity, and an elevated susceptibility to proteolysis [23]. Specifically, mechanical unloading predominantly curtails anabolic signaling [119]. In contrast, systemic pathologies inflict metabolic and inflammatory stress [120]. Meanwhile, aging brings about inherent defects in ribosomal heterogeneity and nucleolar integrity, which collectively exacerbate the disruption of protein homeostasis [121].

This integrated viewpoint highlights a foundational divergence between muscle hypertrophy and atrophy. Specifically, hypertrophic processes are contingent upon mTORC1–c-Myc–orchestrated ribosomal expansion, which facilitates augmented translational capacity and enhanced efficiency [24,28]. Conversely, atrophy is typified by AMPK–FOXO-dependent repression of ribosome biogenesis and concurrent nucleolar disruption [29,122,123]. This stark differentiation not only delineates distinct signaling architectures but also accentuates the ribosome’s role as a molecular pivot point, mediating the delicate balance between muscle growth and wasting [15].

Table 1 additionally consolidates these underlying mechanisms, highlighting common pathways like impaired rRNA transcription and reduced ribosomal protein expression, as well as context-specific features, including inflammatory repression in cachexia and nucleolar fragmentation during aging [17]. This detailed comparative analysis thus underscores ribosomal dysfunction as a consistent, unifying feature spanning various pathological conditions.

**Table 1:** Integrated mechanisms of ribosomal dysfunction in muscle atrophy and related pathologies.

Context	Mechanisms and Features	Clinical Implications	References
Disuse-induced atrophy	Reduced 47S pre-rRNA transcription; downregulation of ribosomal protein genes; suppressed mTORC1–UBF/TIF-1A signaling; AMPK activation; activation of ribophagy (selective ribosome degradation); reduced nucleolar activity (reversible with reloading)	Early biomarker of immobilization-induced atrophy; monitoring ribosome recovery may guide rehabilitation strategies; potential therapeutic target for preventing muscle loss during bed rest or microgravity.	[23]
Disease-associated atrophy	Impaired rRNA transcription and processing; reduced ribosomal protein synthesis; activation of IL-6, TNF-α, STAT3, NF-κB, and FOXO; DNA damage and oxidative stress (chemotherapy); nucleolar disruption; ribosome stress; reduced translational fidelity	Prognostic biomarker in cancer cachexia; therapeutic target for anti-inflammatory or nucleolar-protective interventions; relevant to long-term weakness in CKD, heart failure, ICU patients, and cancer survivors.	[124]
Aging-related atrophy	Blunted rRNA induction after exercise or nutrition; reduced ribosomal protein transcripts; impaired mTORC1 sensitivity; FOXO activation; altered ribosome heterogeneity; fragmented nucleoli; defective rRNA modifications; reduced translational accuracy	Central contributor to sarcopenia; ribosome heterogeneity as a novel therapeutic frontier; diminished responsiveness to standard interventions; potential biomarker for individualized exercise–nutrition strategies.	[22,24]

Note: mTORC1, mechanistic target of rapamycin complex 1; UBF, upstream binding factor; TIF-1A, transcription initiation factor-1A; AMPK, AMP-activated protein kinase; IL-6, interleukin-6; TNF-α, tumor necrosis factor-alpha; STAT3, signal transducer and activator of transcription 3; NF-κB, nuclear factor-kappa B; FOXO, Forkhead box protein O; CKD, chronic kidney disease; ICU, intensive care unit.

From a clinical translation perspective, ribosomal dysfunction is recognized as not merely a mechanistic contributor, but also as a quantifiable biomarker and a viable therapeutic objective [125]. The incorporation of ribosome biology into routine clinical practice holds the potential to facilitate earlier patient risk stratification, the development of individualized intervention plans, and the introduction of innovative therapeutic approaches [19,77]. Such integration effectively connects foundational molecular insights with the practical clinical management of conditions characterized by muscle wasting [126].

## 5. Ribosome Heterogeneity and Selective Translation

In addition to the quantitative regulation of ribosome biogenesis, qualitative distinctions in ribosomal composition and functionality have increasingly been recognized as critical regulatory mechanisms governing translational control [35,127]. The concept of “ribosome specialization” posits that inherent structural variations among individual ribosomes permit the selective translation of specific mRNA subsets [128]. This observed heterogeneity could elucidate why disparate cellular stimuli trigger unique protein synthesis programs, even when global ribosome content remains comparable [36]. Within skeletal muscle, ribosomal heterogeneity may consequently serve as a crucial fine-tuning mechanism, maintaining equilibrium among anabolic growth, adaptive stress responses, and metabolic homeostasis [129].

### 5.1. Structural Basis of Ribosome Specialization

**Figure 4:**
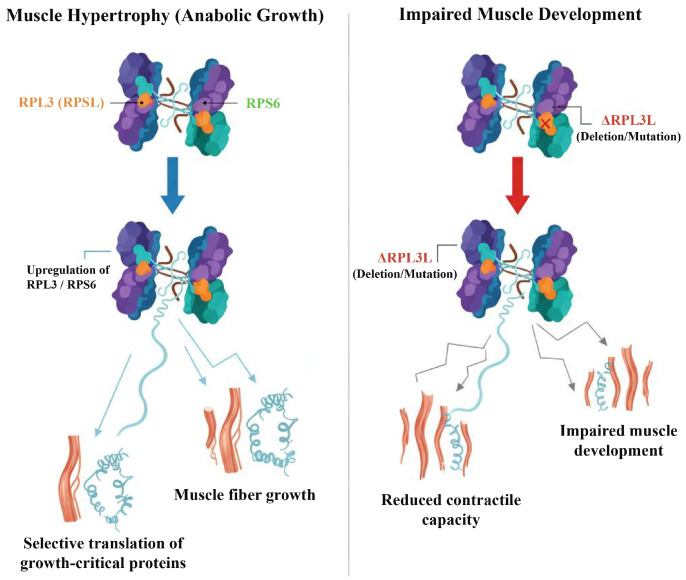
Structural basis of ribosome specialization in skeletal muscle. This figure illustrates how ribosomal protein composition directs selective translation and influences muscle adaptation. (**Left**) During muscle hypertrophy, ribosomes containing specific RPs (RPL3 and RPS6) are upregulated, selectively translating mRNAs critical for growth. (**Right**) In contrast, deletion or mutation of RPL3L disrupts ribosomal function, alters translational dynamics, and reduces expression of contractile proteins, leading to impaired muscle development. Note: RPs, ribosomal proteins; RPL3, Ribosomal Protein L3; RPS6, Ribosomal Protein S6; RPL3L, Ribosomal Protein L3-like; Δ, deletion or mutation.

Proteomic and genetic investigations collectively demonstrate that the integration of particular RPs can influence specific aspects of ribosome function [130,131]. For instance, RPL3 and RPS6 exhibit preferential upregulation during hypertrophic processes, potentially enabling the selective translation of mRNAs critical for muscle growth [132] (Figure 4). Conversely, experimental evidence reveals that the absence or specific mutations of certain RPs can compromise muscle development or its contractile capacity, thereby emphasizing their indispensable functional roles [39,133]. To illustrate, deletion of the ribosomal protein L3-like (RPL3L) paralog within cardiac and skeletal muscle tissue in mice resulted in altered translational dynamics and a marked reduction in contractile protein expression [39] (Figure 4). Furthermore, variations in the ribosomal protein S27-like (RPS27L) promoter region have been shown to impact skeletal muscle growth across various model organisms [134,135]. Taken together, these observations imply that ribosomal heterogeneity is likely not a stochastic event, but rather represents an adaptive mechanism precisely customized to meet diverse physiological requirements.

### 5.2. Post-Translational Modifications and Associated Factors

Beyond variations in RP composition, the functional landscape of ribosomes is further shaped by post-translational modifications of RPs and chemical modifications of rRNA, such as methylation and pseudouridylation [37,136]. These modifications are known to modulate ribosome activity and fidelity [36,137,138,139]. Notably, these modifications undergo dynamic regulation in response to various stress conditions, thereby enabling ribosomes to adapt to shifting metabolic or inflammatory cellular milieus [139,140,141]. Moreover, ribosome-associated factors, including various RNA-binding proteins and microRNAs, can interact directly with ribosomes, influencing transcript selectivity [142,143,144]. Collectively, these multifaceted mechanisms endow ribosomes with intricate regulatory control extending well beyond their fundamental role in protein synthesis [36,142].

### 5.3. Functional Relevance in Skeletal Muscle Plasticity

Despite the well-recognized phenomenon of ribosome heterogeneity across various tissues, its precise functional implications within skeletal muscle are not yet fully elucidated [129]. Evidence from animal models suggests that a selective ribosomal function might play a role in the preferential translation of structural proteins during muscle hypertrophy, or stress-protective proteins during atrophy [37]. Nevertheless, human investigations into this area are limited, primarily owing to difficulties in tissue accessibility and inherent technical complexities [145,146]. Promising advances in methodologies, including ribosome profiling, cryo-electron microscopy, and single-fiber analyses, are anticipated to shed light on whether ribosome heterogeneity actively drives or merely correlates with muscle adaptation [129].

## 6. Therapeutic Perspectives: Exercise, Nutrition, and Pharmacology

Mechanistic understandings derived from ribosome biology have laid a crucial groundwork for developing therapeutic strategies aimed at preserving or enhancing skeletal muscle mass [3]. These interventions broadly include exercise training, nutritional supplementation, and various pharmacological agents [33,147]. Notably, these diverse approaches ultimately converge on the shared objective of optimizing ribosome biogenesis and enhancing overall translational efficiency [148,149,150,151].

### 6.1. Exercise Training

RE stands as the most potent non-pharmacological inducer of ribosome biogenesis [150]. Acute bouts of RE have been shown to elevate 47S pre-rRNA transcription within hours [25,152], whereas sustained ribosome accumulation and subsequent hypertrophy are hallmarks of chronic resistance training [25]. In contrast, endurance exercise primarily drives mitochondrial biogenesis, with only modest effects observed on ribosomal pathways [42,153]. Interestingly, concurrent training, which integrates both resistance and endurance stimuli, may yield synergistic adaptations by coordinately promoting ribosome and mitochondrial expansion [154,155,156,157]; however, the precise balance of these effects is contingent upon training volume and intensity [122,150]. From a clinical perspective, resistance training serves as a foundational therapy for sarcopenia, underscoring its significant translational relevance [158,159].

### 6.2. Nutritional Strategies

Nutritional interventions, especially dietary protein supplementation, have been shown to act synergistically with exercise in stimulating ribosome biogenesis [150]. Leucine, a key branched-chain amino acid, is particularly effective at potently activating mTORC1 signaling and subsequently enhancing rRNA transcription [149,160]. Human research indicates that high-protein diets improve anabolic responses in both younger and older populations, with optimal protein distribution throughout the day further enhancing ribosome-related pathways [161]. Furthermore, accumulating evidence suggests that protein quality, for instance, distinctions between whey and plant-based proteins, differentially influences ribosomal signaling, holding significant implications for clinical nutritional strategies in managing sarcopenia and cachexia [161,162].

### 6.3. Pharmacological Interventions

Pharmacological strategies aimed at modulating ribosome biogenesis represent an emerging, albeit challenging, area of therapeutic development [163]. Although direct activators of anabolic signaling pathways, such as mTORC1 agonists or c-Myc activators, have been proposed to restore ribosome production during conditions of catabolic stress [164], their clinical application faces significant hurdles due to considerable oncogenic potential and unresolved long-term safety concerns [165]. The strong association between excessive ribosome activation and uncontrolled cell proliferation [166] critically underscores the imperative for a carefully balanced therapeutic approach in this domain.

An alternative therapeutic strategy involves the indirect modulation of ribosome function through interventions designed to mitigate catabolic signaling or preserve nucleolar integrity [167,168]. For instance, anti-inflammatory agents, such as IL-6/STAT3 inhibitors, and various antioxidants, including N-acetylcysteine and resveratrol, have demonstrated promise in preclinical and early clinical investigations. These compounds appear to alleviate ribosome stress and partially restore protein synthesis capacity [169]. Similarly, metabolic modulators like metformin and beta-adrenergic agents may exert secondary beneficial effects on ribosomal pathways by reducing systemic inflammation and enhancing cellular energy availability [170]. These collective observations suggest that maintaining ribosome homeostasis via indirect pharmacological approaches could offer a safer and more translationally feasible route compared to direct anabolic stimulation [169].

In summary, although the pharmacological activation of ribosome biogenesis presents a conceptually appealing therapeutic avenue, its clinical translation necessitates a thorough risk–benefit evaluation [171]. The most promising immediate applications likely reside in combination strategies, wherein exercise and nutritional interventions serve as the primary anabolic drivers, with pharmacological agents functioning as supportive modulators to ameliorate catabolic stress and sustain ribosomal competence [169].

### 6.4. Clinical Translation and Ongoing Trials

Clinical evidence supporting strategies that specifically target ribosome function is currently emerging, though still somewhat limited [18]. Among existing interventions, the combination of resistance training and protein supplementation offers the most robust support: randomized controlled trials involving older adults with sarcopenia consistently demonstrate improvements in muscle mass and strength, which are partly attributable to enhanced ribosomal signaling and protein synthesis [172,173]. Nutritional strategies, particularly those incorporating leucine-enriched or optimized protein diets, effectively activate mTORC1 and stimulate translational initiation [174,175,176,177]; however, their efficacy can be diminished in elderly populations due to anabolic resistance [161,178]. Pharmacological approaches, by contrast, largely remain in exploratory stages [179]. Indirect agents, such as certain antioxidants and metabolic modulators, have shown preliminary benefits [180], while direct ribosome activators are currently restricted to preclinical models owing to persistent safety concerns [19].

These findings are comprehensively summarized in Table 2, which details representative interventions, their underlying ribosome-related mechanisms, and the current level of supporting evidence. Broadly, exercise and nutritional strategies have advanced to human clinical trials, yielding encouraging, though somewhat variable, outcomes [181]. In contrast, pharmacological options are predominantly in earlier stages of investigation [25,150,182]. Future clinical translation efforts should prioritize high-risk populations, including older adults afflicted with sarcopenia, cancer survivors experiencing cachexia, and critically ill patients suffering from disuse atrophy [183]. Crucially, the concurrent development of minimally invasive biomarkers of ribosome function will be indispensable for effective patient stratification, optimized treatment monitoring, and the facilitation of precision interventions [184,185]. These considerations collectively establish the context for the broader perspectives explored in the subsequent section.

**Table 2:** Therapeutic strategies targeting ribosome function in muscle atrophy: mechanisms and evidence.

Therapeutic Approach	Key Mechanisms	Evidence & Clinical Translation	References
Exercise training	RE: Stimulates ribosome production and muscle growth. Endurance exercise: Primarily enhances mitochondrial biogenesis with limited impact on ribosome function.	Strong evidence: Proven effective in improving muscle mass and strength, widely recommended for sarcopenia.	[25,150]
Nutritional strategies	Protein supplementation: Enhances ribosome biogenesis, especially with leucine. Protein quality: Different proteins (whey vs. plant) affect ribosomal signaling.	Well-supported: High-protein, leucine-enriched diets activate mTORC1, beneficial for sarcopenia and cachexia.	[174]
Pharmacological interventions	Direct activators (e.g., mTORC1 agonists, c-Myc activators): Stimulate ribosome production during catabolic stress. Indirect modulators (e.g., anti-inflammatory, antioxidants): Preserve nucleolar integrity and reduce ribosome stress.	Preclinical and early clinical trials: Direct activators face safety concerns. Indirect agents (e.g., antioxidants, metformin) show promise, with safer profiles.	[19,22]

Note: mTORC1, mechanistic target of rapamycin complex 1; c-Myc, cellular myelocytomatosis oncogene.

## 7. Emerging Questions and Future Directions

Notwithstanding the rapid advancements made in the field, several critical gaps persist within ribosome biology, particularly concerning its application to skeletal muscle adaptation [3,186]. Addressing these pivotal questions will be indispensable for enhancing translational relevance and propelling this field closer to tangible clinical applications.

### 7.1. Coupling of Ribosome Biogenesis and Mitochondrial Function

Ribosome and mitochondrial biogenesis represent highly interdependent cellular processes [43,79]. Ribosomes are responsible for synthesizing the nuclear-encoded proteins essential for mitochondrial function, while mitochondria, in turn, supply the necessary adenosine triphosphate for ribosome assembly [19,187]. For instance, investigations have revealed that resistance training primarily induces ribosomal content expansion, whereas endurance training predominantly promotes mitochondrial proliferation [25,153,188]. Nevertheless, the precise molecular crosstalk governing the interaction between these two critical systems remains largely undefined. Unraveling how distinct exercise modalities or specific pharmacological agents differentially influence this intricate interaction will be crucial for developing interventions that optimize both anabolic and metabolic adaptations [79,187,189]. Significantly, this complex inter-organellar dialogue may also dictate the equilibrium between muscle hypertrophy and endurance capacity [79,189], thereby directly impacting exercise prescription strategies.

### 7.2. Ribosome Heterogeneity as a Therapeutic Target

The intriguing concept of ribosome heterogeneity, characterized by variations in ribosomal protein composition or rRNA modifications, presents the compelling possibility of precisely tailoring translational programs to meet specific cellular demands [190]. Initial studies indicate that these heterogeneous ribosomes can selectively translate particular subsets of mRNAs, thereby enabling a fine-tuning of adaptive responses [191,192,193]. However, compelling evidence supporting this phenomenon in human skeletal muscle remains limited [129]. Consequently, the development of small molecules or gene-editing tools capable of selectively modulating ribosomal composition could unveil novel therapeutic avenues [194,195,196,197]. Nonetheless, rigorous validation within preclinical disease models is imperative to establish both the safety and feasibility of such approaches before their progression to clinical trials.

### 7.3. Disease Cohorts for Precision Interventions

A significant challenge in translating ribosome-centered strategies into clinical practice lies in precisely identifying patient populations most likely to benefit [19]. Priority cohorts for investigation include cancer survivors afflicted with cachexia, critically ill ICU patients experiencing rapid disuse atrophy, and older adults suffering from sarcopenia. These groups share a high burden of anabolic resistance [198,199] and frequently exhibit molecular signatures indicative of impaired ribosome biogenesis or reduced translational capacity [200]. Consequently, pilot studies conducted within these populations could offer critical insights into the therapeutic potential of ribosome-targeted interventions, a notion supported by recent mechanistic and translational reviews focusing on cachexia and critical illness [6,199]. Furthermore, stratifying patients based on robust biomarkers of ribosome function, such as 47S pre-rRNA/rDNA transcription levels, total RNA content as an indicator of ribosome content, ribosome profiling-derived translational readouts, or specific rRNA epitranscriptomic marks, may prove instrumental in personalizing treatment regimens and maximizing therapeutic benefits [201].

### 7.4. Biomarker Development and Detection Methods

A notable impediment to clinical application is the current dearth of minimally invasive biomarkers specifically for ribosome biogenesis [202]. While muscle biopsies are considered the gold standard for assessing ribosomal status, their invasiveness renders them impractical for routine clinical monitoring [203]. Promising alternative strategies are emerging, including peripheral blood markers such as circulating ribosomal protein fragments or indicators of nucleolar stress [204,205]. Furthermore, advanced imaging techniques, particularly positron emission tomography tracers designed to target ribosome activity [206,207], alongside multi-omics approaches that integrate ribosome profiling with proteomics and metabolomics [208,209], offer potential solutions. Such innovations could facilitate the early detection of anabolic resistance, enable more precise patient stratification, and allow for individualized monitoring of therapeutic efficacy [210,211].

Moving forward, future research endeavors should focus on bridging fundamental molecular mechanisms with clinically applicable strategies. This involves several key objectives: (i) elucidating the intricate crosstalk between ribosomes and mitochondria [212,213,214]; (ii) rigorously validating the phenomenon of ribosome heterogeneity within human muscle tissue [129]; (iii) prioritizing investigations in high-risk patient cohorts [118]; and (iv) developing robust, minimally invasive biomarkers [204,206]. Such concerted efforts will be instrumental in laying the foundation for precision ribosome-targeted interventions aimed at preserving muscle mass and promoting functional health across a wide spectrum of clinical contexts [215].

## 8. Conclusions

Skeletal muscle plasticity is fundamentally constrained by ribosome biogenesis and translational control. Across diverse atrophic conditions, including disuse, aging, chronic disease, and cancer cachexia, suppression of ribosome production and impaired translational efficiency consistently emerge as early and unifying molecular features. In contrast, sustained muscle hypertrophy requires robust expansion of the ribosomal pool, underscoring ribosome biogenesis as a rate-limiting determinant of muscle mass regulation rather than a passive downstream process.

Beyond ribosome quantity, accumulating evidence highlights ribosome heterogeneity as a qualitative layer of translational regulation in skeletal muscle. Variations in ribosomal protein composition and rRNA modifications may enable selective translation of mRNA subsets during muscle growth, stress adaptation, and degeneration. Although this concept remains incompletely characterized in human muscle, it represents a promising mechanistic frontier for understanding anabolic resistance and context-specific muscle responses.

From a translational perspective, ribosome biology provides a conceptual bridge between molecular mechanisms and therapeutic strategies. Exercise training and nutritional interventions remain the most effective approaches to preserve ribosomal competence, while indirect pharmacological strategies targeting inflammation, metabolic stress, and nucleolar integrity may offer complementary benefits. Importantly, ribosome-related readouts, such as rRNA transcriptional activity and translational efficiency, hold potential as biomarkers for early risk stratification and precision intervention in muscle-wasting disorders.

In summary, this review positions ribosome biogenesis and translational control as central regulatory nodes in skeletal muscle adaptation. By shifting focus from upstream signaling alone to ribosome-centered regulation, future research may enable more precise, mechanism-based strategies to preserve muscle mass, improve functional outcomes, and promote healthy aging.

## Data Availability

All relevant materials are presented in this manuscript.
